# Auto-Abs against type I IFNs: Strong, common, and global determinants of severe arboviral diseases

**DOI:** 10.70962/jhi.20250090

**Published:** 2025-10-23

**Authors:** Adrian Gervais, Alessandro Borghesi, Jean-Laurent Casanova, Shen-Ying Zhang

**Affiliations:** 1 Laboratory of Human Genetics of Infectious Diseases, Necker Branch, Institut National de la Santé et de la Recherche Médicale (INSERM) U1163, Necker Hospital for Sick Children, Paris, France; 2 Paris Cité University, Imagine Institute, Paris, France; 3 Host-Pathogen Group, San Matteo Research Hospital, Pavia, Italy; 4 Neonatal Intensive Care Unit, San Matteo Research Hospital, Pavia, Italy; 5 Mother, Child and Adolescent Department, Neonatal Intensive Care Unit, Geneva University Hospitals, Geneva, Switzerland; 6 Department of Pediatrics, Gynecology and Obstetrics, Faculty of Medicine, University of Geneva, Geneva, Switzerland; 7 School of Life Sciences, Swiss Federal Institute of Technology, Lausanne, Switzerland; 8 St. Giles Laboratory of Human Genetics of Infectious Diseases, Rockefeller Branch, Rockefeller University, New York, NY, USA; 9 Howard Hughes Medical Institute, New York, NY, USA; 10 Department of Pediatrics, Necker Hospital for Sick Children, Assistance Publique – Hôpitaux de Paris, Paris, France

## Abstract

Human-tropic pathogenic arboviruses are spreading worldwide. There is immense interindividual clinical variability following infection with any arbovirus. Autoantibodies (auto-Abs) neutralizing antiviral type I IFNs (AAN-I-IFN) can underlie a small but growing number of severe arboviral diseases, whether transmitted by ticks (tick-borne encephalitis virus, TBEV; Powassan virus, POWV) or mosquitoes (West Nile virus, WNV; Usutu virus, USUV; Ross River virus, RRV) and whether due to flaviviruses (WNV, TBEV, POWV, and USUV) or alphaviruses (RRV). Evidence is documented in large cohort studies for WNV and TBEV. They can also account severe adverse reactions to the live-attenuated yellow fever virus vaccine. AAN-I-IFN are present before arboviral infection and are the cause of severe disease. Carriers of these auto-Abs are common worldwide (>100 million people), have a very high risk of severe disease (relative risk >100), and account for a sizeable proportion of cases (typically >10%). Other severe diseases due to different arboviruses may also be caused by these auto-Abs.

## Teaser

Autoantibodies neutralizing antiviral type I IFNs (AAN-I-IFN) underlie a small but growing number of severe arboviral diseases. These auto-Abs are strong, common, and global determinants of severe arboviral diseases. Genetic defects of type I IFN immunity may underlie other unexplained cases.

## Introduction

Arthropod-borne viruses, generally abbreviated to arboviruses, form a large group of viruses transmitted to humans by hematophagous vectors such as mosquitoes, ticks, midges, and sandflies (1, 2, 3, 4). They are predominantly ribonucleic acid viruses (among which most are positive single-stranded viruses, and fewer are negative or double-stranded viruses), and rarely deoxyribonucleic acid viruses (a single virus). Key arbovirus families include *Flaviviridae*, *Togaviridae*, *Bunyavirales*, and *Reovirales*. Over 150 arboviruses can cause disease in humans (5) (https://ictv.global, https://wwwn.cdc.gov/arbocat/), and at least half of all human pathogenic viruses are arboviruses (6, 7). The arboviruses with the greatest impact on public health include dengue virus (DENV), chikungunya virus (CHIKV), Japanese encephalitis virus (JEV), yellow fever virus (YFV), Zika virus (ZIKV), West Nile virus (WNV), Oropouche virus, and tick-borne encephalitis virus (TBEV) (3, 8, 9, 10, 11, 12, 13). Arboviral infections have diverse geographic distributions associated with the distributions of their vectors, which vary with climatic, ecological, and urban conditions. For instance, mosquitoes from the *Aedes* and *Culex* genera are the primary vectors for DENV, ZIKV, and WNV, whereas ticks from the *Ixodidae* family transmit TBEV and POWV. The increasing spread of arboviral infections has been attributed partly to climate change and globalization, which facilitate the spread of vector populations (14, 15). In addition, increases in global mobility and trade have facilitated the transportation of infected animals, vectors, and humans. Consequently, arboviruses have become a significant global health burden, as suggested by recent epidemiological studies estimating that 73% of all newly identified human infections are caused by arboviruses (16), which account for infections in at least 400 million people annually (3, 17). Most infections are asymptomatic or benign, and both the frequency and nature of clinical disease differ between viruses, but, overall, about 1/100 infected individuals develop severe, sometimes fatal disease (18). Severe diseases are typically observed more frequently in low-resource regions lacking a robust healthcare infrastructure (19). Arboviruses have a major impact on health and the economy in affected communities, and research is urgently required to find ways to prevent further spread and to reduce disease burden through targeted public health measures and novel therapeutic approaches.

The immense interindividual clinical variability observed following arbovirus infection is similar to that observed with many other pathogens, and the root cause of severe disease remains largely unexplained (7). Epidemiologically, age is the strongest known predictor of neuroinvasive arboviral disease and death, with disease incidence peaking in children and the elderly (e.g., JEV and CHIKV encephalitis) or in the elderly (e.g., WNV, TBEV, and St. Louis encephalitis virus encephalitis) (20, 21). The age-dependent “U-shape” prevalence curve for life-threatening infections is suggestive of inborn errors of immunity (IEI) in childhood or their phenocopies in the elderly, and the “J-shape” curve is suggestive of phenocopies only (22). Type I interferons (IFNs) have the capacity to limit the replication of many viruses, including arboviruses (e.g., WNV, DENV, YFV, and ZIKV), as shown in previous in vitro experimental models using cultured human cell lines (23), and in vivo in murine models (24, 25). In humans, candidate gene-based association studies have attempted to evaluate the contribution of common polymorphisms of certain type I IFN-inducible (*OAS1*, *OASL*, *IRF3*, *MX1*) and other (*HLA* loci, *CCR5*) genes to the outcome of WNV infection in humans. The odds ratios (ORs) ranged from 0.19 to ∼10 (26, 27, 28, 29). However, the reproducibility of most of these data was low, probably due to the small numbers of patients studied or the small effect sizes of the variants studied. The most convincing data were obtained for homozygous CCR5 c.554_585del (OR = 4.4 for symptomatic infection and 13.2 for death), which increases disease severity following infection with WNV (30). In this context, the much higher risk of arbovirus encephalitis in men over the age of 65 years is reminiscent of the pattern observed for critical coronavirus disease 2019 (COVID-19) pneumonia (31), 15–20% of cases of which are due to preexisting circulating autoantibodies (auto-Abs) neutralizing type I IFNs (AAN-I-IFN) (32, 33, 34, 35, 36, 37, 38, 39, 40, 41, 42, 43, 44, 45, 46, 47, 48, 49, 50, 51, 52, 53, 54, 55, 56, 57, 58, 59, 60, 61, 62, 63, 64, 65, 66, 67, 68). Type I IFNs were originally identified and have since been extensively studied as antiviral molecules (69, 70, 71). The type I IFNs are comprised of 13 IFN-α subtypes, one IFN-β, one IFN-ω, one IFN-ε, and one IFN-κ. IFN-ω, IFN-ε, and IFN-κ are, respectively, produced by leukocytes, cells of the female reproductive tract, and keratinocytes, whereas IFN-α and IFN-β are produced by a broader range of human cells. These 17 known subtypes of human type I IFNs all signal through the same receptor composed of IFN-α/β receptor (IFNAR)1 and IFNAR2, which are ubiquitously expressed across the human body. No underlying IEI, related to type I IFNs or not, have yet been reported for arboviral diseases. The AAN-I-IFN occur in the general population (33, 72, *Preprint*) and underlie at least two other types of critical viral pneumonia (73, 74). We review recent findings indicating that in every country where they have been searched for, AAN-I-IFN are strong and common determinants of a growing number of severe arboviral diseases.

## Severe adverse reaction to live-attenuated YFV vaccine

YFV is an orthoflavivirus of the *Flaviviridae* family that is mainly viscerotropic but can also exhibit neurotropic properties (75). It circulates principally in Africa and Central and South America. It can be transmitted to humans by *Aedes* and *Haemagogus* mosquitoes and results in life-threatening disease in about one third of cases (76). YFV infection triggers ∼200,000 cases of disease and 30,000 deaths annually worldwide, with 90% of cases occurring in Africa (77). The live-attenuated YFV-17D vaccine has been widely available since 1938 and is considered effective and safe (78, 79, 80). However, very rare cases of severe adverse reaction to YFV-17D vaccine due to uncontrolled viral replication have occasionally been reported (81, 82, 83, 84, 85). In 2019, we reported a 14-year-old girl with autosomal recessive (AR) IFNAR1 deficiency and no prior history of severe viral illness who developed severe viscerotropic disease following YFD-17D vaccination, highlighting the fundamental role of type I IFNs in controlling YFV-17D (86). In this context, we recently studied seven other previously healthy patients aged 13–80 years with unexplained life-threatening YFV vaccine–associated disease (four with viscerotropic disease, two with neurotropic disease, and one with both) (87). One 13-year-old patient was found to have AR complete IFNAR2 deficiency. Three of the remaining six patients, vaccinated at the ages of 47, 57, and 64 years, had circulating auto-Abs neutralizing IFN-α and IFN-ω, and two of these patients also had auto-Abs neutralizing IFN-β. The binding capacity of these auto-Abs extended to all the other type I IFN subtypes except IFN-κ and IFN-ε, as observed in severe COVID-19 cases. Five of the eight patients (∼60%) studied by our group therefore had insufficient type I IFN immunity, explained in three of the eight (∼37.5%) patients by the presence of auto-Abs against type I IFNs. Consistently, another group found auto-Abs recognizing type I IFNs in three of the 10 patients (30%) studied (88); the neutralization capacity of these auto-Abs was not assessed. Overall, these results demonstrate that genetic defects of type I IFN immunity or auto-Abs neutralizing type I IFNs can underlie severe YFV-17D infection. These data also suggest that patients with known type I IFN deficiencies should, thus, avoid YFV vaccination, and pre-vaccination screening for auto-Abs could be useful. Moreover, naturally occurring YFV infection may itself be worsened by type I IFN deficiency and especially by AAN-I-IFN.

## WNV neuroinvasive disease

**Figure 1.**
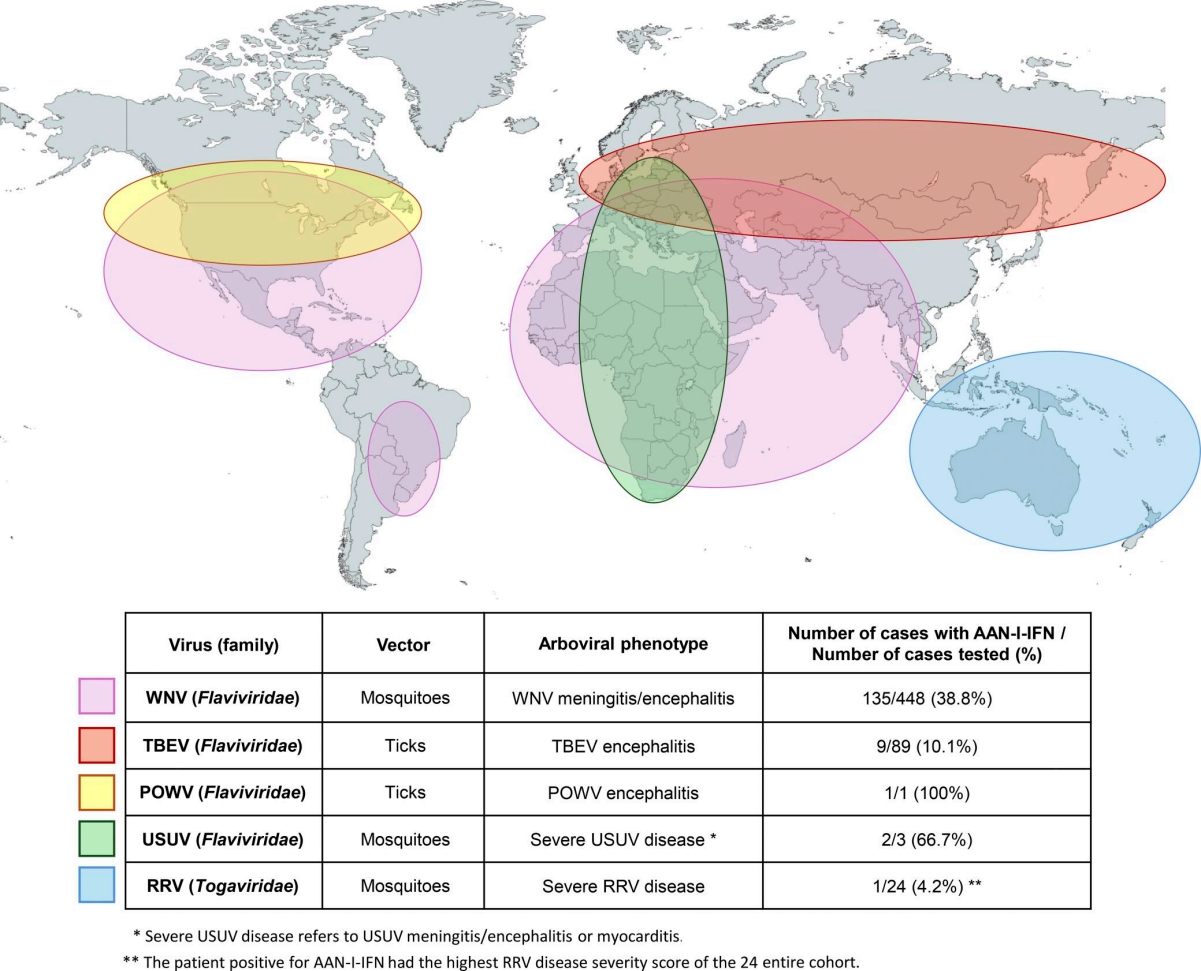
Map of the distribution of the most endemic regions of the arboviruses for which auto-Abs against type I IFNs have been shown to underlie severe disease.

WNV is a neurotropic mosquito-borne orthoflavivirus of the *Flaviviridae* family. Outbreaks of WNV infection are increasing in frequency and magnitude, and the geographic range of this virus is continually increasing worldwide (89, 90, 91, 92, 93). Infection with WNV triggers encephalitis in about 1 in 150 infected individuals, although this prevalence may be underestimated (94, 95, 96, 97, 98). In 2023, we investigated an international cohort of 663 individuals infected with WNV, including 114 with silent infection, 104 with ambulatory mild infection (West Nile fever), and 441 hospitalized for WNV disease (WNVD), including 348/441 with neuroinvasive disease (encephalitis/meningitis). We found that ∼35% of the patients with WNVD had auto-Abs against IFN-α and/or IFN-ω, rising to ∼40% among those with WNV encephalitis, the most severe manifestation of WNV infection (99) (Fig. 1). By contrast, only 1.8% of individuals with silent infection had auto-Abs, a proportion similar to that in the general population. Individuals with auto-Abs neutralizing low concentrations of IFNs (100 pg/ml, about 10 times of the physiological circulating I-IFN concentration in the human body) had an ∼20 times higher risk of WNVD, and those with auto-Abs neutralizing high concentrations of IFNs (10 ng/ml) had a 130 times higher risk of WNVD than individuals without such auto-Abs. This risk was even higher in people under the age of 65 years, with an OR of about 500. Moreover, these auto-Abs were present in the cerebrospinal fluid of about 70% of patients with detectable systemic auto-Abs. Importantly, our data indicate that AAN-I-IFN are present before WNV infection and that their levels remain stable over time, as they remain detectable months after the infection, consistent with the findings of a longitudinal study of Swiss patients with HIV infection (72, *Preprint*). A subsequent study reported life-threatening WNV infection in a patient with thymoma who also carried highly neutralizing auto-Abs against IFN-α and IFN-ω and was homozygous for CCR5 c.554_585del (100). These auto-Abs against IFN-α, -β, or -ω impair the protective anti-WNV response of the corresponding type I IFNs in VERO-E6 and ARPE-19 cells in vitro (99, 101). Moreover, the blockade of I-IFN signaling led to increased infection of mouse enterocytes and gut and blood–brain barrier permeability changes in mice in vivo, resulting in more severe disease (102). Consistently, blockade of I-IFN signaling by human AAN-I-IFN led to increased WNV infection in human enteroid cultures. Finally, AAN-I-IFN have recently been found in two out of the three very rare cases studied with severe disease following infection by Usutu virus (USUV) (103), another mosquito-borne orthoflavivirus closely related to WNV, probably via similar mechanisms. Overall, AAN-I-IFN are causal for WNV encephalitis in about 40% of cases, which makes WNV encephalitis the human infectious disease for which the underlying mechanism is best explained to date.

## Tick-borne encephalitis

TBEV is another neurotropic orthoflavivirus of the *Flaviviridae* family. It spreads mainly in Europe and Asia. It is transmitted to humans primarily via the bite of infected ticks, typically *Ixodes ricinus* and *Ixodes persulcatus*, which serve as both vector and reservoir hosts of the European and the Asian subtypes of TBEV. More rarely, TBEV can be transmitted via unpasteurized milk or dairy products from cattle exposed to ticks in endemic areas. TBEV infection is benign in at least 90% of cases but affects the central nervous system (CNS) in the remaining cases, causing mild (<5%), moderate (<4%), or severe (<1%) disease (2, 104, 105, 106, 107). We studied a cohort of 441 individuals infected with TBEV from Austria, the Czech Republic, France, and Italy: 174 mild (meningitis), 178 moderate (meningitis and one CNS lesion), and 89 severe (TBEV encephalitis [TBE] with multiple CNS lesions) cases (108). We found that AAN-I-IFN were present in about 10% of the 89 TBE cases, versus only about 1% of the moderate and mild cases. None of the 13 asymptomatic cases had such auto-Abs. Auto-Abs capable of neutralizing high concentrations (10 ng/ml) of type I IFNs conferred a significantly higher risk of TBE, with an OR of ∼21 for patients with auto-Abs neutralizing both IFN-α2 and IFN-ω. Auto-Abs neutralizing IFN-ω alone, at lower concentrations (100 pg/ml), also markedly increased the risk, with an OR of ∼7. Mortality was 25% in TBE cases with auto-Abs, versus only 7.5% in those without auto-Abs. As observed in the WNV study, younger patients (<65 years old) with auto-Abs had a higher estimated risk of severe disease than older patients (OR of ∼26 vs. ∼4) because auto-Abs are rarer in the <65 years age group of the general population. The actual risk may be higher in older individuals, whose type I IFN immunity is declining for other reasons, such as the apparent decreasing type I IFN production or response ability of some blood leukocytes (109, 110, 111). Auto-Ab levels remained stable over time in the few longitudinal samples tested. These auto-Abs were able to block the protective effect of exogenous IFN-α2 and IFN-ω against TBEV infection in Vero-E6 cells in vitro. These findings underscore the pathogenic role of AAN-I-IFN in the development of TBE. It is noteworthy that AAN-I-IFN have been found also in an extremely rare case of encephalitis following infection by Powassan virus (POWV), another tick-borne orthoflavivirus (103), suggesting a more general role of AAN-I-IFN in the development of encephalitis triggered by these tick-borne orthoflaviruses (Fig. 1). This hypothesis warrants further investigation. The finding of AAN-I-IFN in patients with WNV encephalitis or TBE, in turn, also suggests that the extremely rare cases of WNV encephalitis or TBE in patients with deleterious mutations of *GATA2* or *IRF7* are probably also due to impaired type I IFN antiviral immunity (112, 113).

## POWV encephalitis

POWV is a tick-borne neurotropic orthoflavivirus of the *Flaviviridae* family endemic to North America that can cause serious neurological infections, such as encephalitis, particularly in older individuals (2, 114, 115). It can be transmitted to humans by the bites of *Ixodes* ticks, typically *Ixodes cookei*, *Ixodes marxi*, or *Ixodes scapularis*. Since the first human cases identified in 1958 in Ontario, Canada, and in 1970 in New Jersey in the United States, more than 250 cases have been reported over the years in Canada and the United States (115). Between 2004 and 2015, typically fewer than 10 cases were reported in the United States annually, but this number now ranges between 20 and 50 in recent years (CDC, https://www.cdc.gov/powassan/data-maps/historic-data.html#cdc_data_surveillance_section_4-view-the-historic-data). However, the prevalence of POWV infection in North America is unknown; it is probably well above the number of reported cases. Individuals without neuroinvasive symptoms are rarely tested, if at all, so very little is known about subclinical or mild infection (115). We studied three North American patients with POWV disease: two men aged 37 and 70 years hospitalized with moderate disease and resulting in almost complete recovery, and a 68-year-old woman who developed severe encephalopathy, progressing to chronic respiratory failure and requiring ventilation support until her death a year later due to long-term sequelae (103). The two moderate cases had no detectable AAN-I-IFN, whereas the severe case had high levels of auto-Abs neutralizing both high and low concentrations of IFN-ω. All the samples tested were obtained from the patients in the first few days after infection. It is important to assess AAN-I-IFN in more patients with POWV infection, but this severe case already suggests that preexisting auto-Abs targeting type I IFNs probably play a role in the development of severe POWV infection. POWV is the second tick-borne neurotropic orthoflavivirus, after TBEV, for which AAN-I-IFN have been shown to result in severe infection (Fig. 1). These findings suggest that other patients suffering from severe TBEV or POWV infections, or other tick-borne viral diseases, should be tested for the presence of AAN-I-IFN and for genetic defects of type I IFN immunity.

## Severe USUV disease

USUV is a mosquito-borne orthoflavivirus of the *Flaviviridae* family endemic to Africa and Europe (116, 117, 118, 119, 120). It was first identified in field-caught *Culex neavei* mosquitos in South Africa in 1959 and then in other African countries shortly afterward. The virus is thought to have been introduced into Europe in the 1960s and has already spread to other regions, including the Middle East and North America (121, 122). Cases of human USUV disease were first reported in Africa in the 1980s (116, 117, 118, 119, 120), then about 20 years later in Europe (116, 123). Those two regions account for all reported human cases so far, a total of between 200 and 300 cases, mostly in Italy and Austria (122, 123, 124, 125, 126). USUV typically causes asymptomatic infections in humans. However, some immunocompromised individuals may display severe manifestations, mostly neurological. Unfortunately, the prevalence of USUV in Africa or Europe is unknown, and the acute USUV infections actually reported are not representative of the full spectrum of human USUV infections, as virus studies have generally been limited to cohorts of patients with signs of neurological infections of various degrees of severity or accidentally identified via donated blood samples. In a recent study, 34 USUV-infected individuals were tested for the presence of AAN-I-IFN in the blood: 31 asymptomatic cases and three severe cases (103). The three symptomatic cases, all men, aged 43, 78, and 80 years, displayed manifestations ranging from meningitis to myocarditis and meningoencephalitis, with one patient succumbing to the infection. Auto-Abs neutralizing both high and low concentrations of IFN-α2, IFN-ω, and IFN-β were detected in two of the severe cases. One had USUV meningitis and the other had severe myocarditis, leading to cardiogenic shock. The asymptomatic cases had no such auto-Abs, underscoring the link between the presence of neutralizing auto-Abs and the progression to severe USUV disease (Fig. 1). These findings suggest that preexisting neutralizing auto-Abs against IFNs predispose individuals to severe manifestations of USUV infection. It would be important to assess the presence of AAN-I-IFN in the blood of other cases of severe USUV infection.

## Severe Ross River virus (RRV) disease

RRV is an alphavirus of the *Togaviridae* family. It is prevalent in Australia and other parts of Oceania, with mosquito transmission contributing to annual outbreaks (127, 128, 129). RRV was first isolated from an *Aedes vigilax* mosquito around 1960 near the Ross River in Australia (https://www.health.vic.gov.au/infectious-diseases/ross-river-virus-disease), about 40 years after the first documented outbreak of RRV disease (also known as epidemic polyarthritis) in 1928 in Australia (127). A wide variety of mosquito species have since been found to transmit RRV. Unlike WNV, TBEV, POWV, and USUV, all of which are neurotropic flaviviruses, RRV is an alphavirus with an arthritogenic rather than neurotropic tropism. Infected individuals do not usually require hospitalization, and symptomatic cases are generally self-limiting, with symptoms and signs including fever and polyarthritis. Approximately 5,000 cases of RRV disease are recorded in Australia annually, making this condition the most common mosquito-borne disease in Australia. One recent study analyzed 96 individuals infected with RRV and with a disease severity ranging from mild to severe (103). Severe cases were defined as high clinical severity scores derived from a multidimensional reduction of the severity of prevalent manifestations (e.g., restless sleep and extended sick leave) (130). None of the individuals studied were hospitalized. Samples from one of the 24 patients with severe disease, a 55-year-old woman, neutralized high and low concentrations of IFN-α2. This patient had the highest severity score of the entire cohort and, thus, the most severe disease of any of the patients tested. Interestingly, this patient was the only patient to report both headaches and fever during the entire course of infection, suggesting perhaps an unusual neurotropism of RRV. Her auto-Ab levels were stable as her blood continued neutralizing type I IFNs when tested a year later, whereas the blood of other mild and moderate cases displayed no type I IFN-neutralizing activity when tested postinfection. These results suggest that, although RRV rarely leads to severe disease, preexisting AAN-I-IFN may increase the severity of disease following infection (Fig. 1). Other cohort studies are warranted.

## Concluding remarks

**Figure 2.**
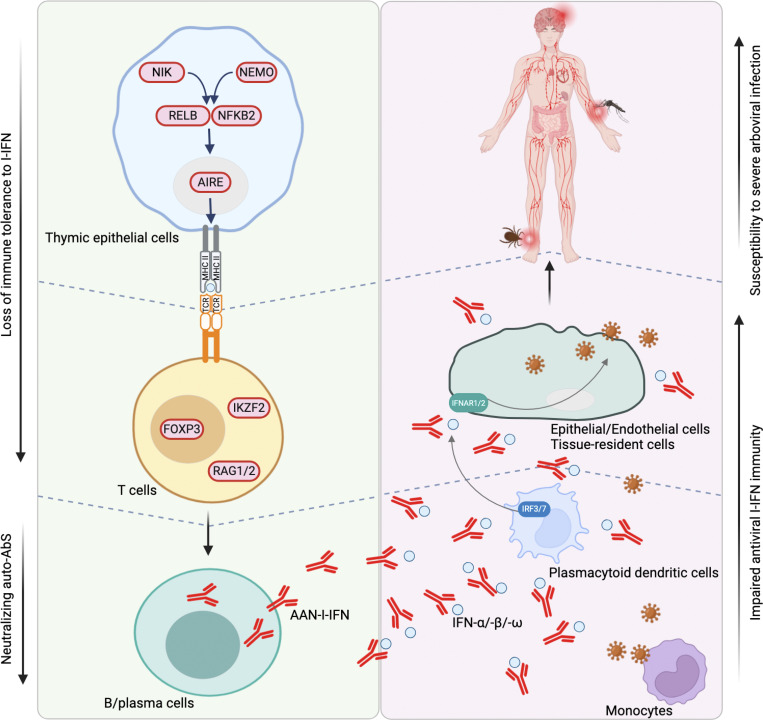
**The human genetic causes of AAN-I-IFN and their consequences.** Molecules shaded in pink color are known human genetic defects underlying thymic T cell tolerance to self (I-IFN) that result in the generation of AAN-I-IFN. The AAN-I-IFN impair I-IFN–mediated antiviral immunity in the peripheral and specific organs, including the brain, thereby underlying severe arboviral diseases. This figure was created using the BioRender app (https://biorender.com).

AAN-I-IFN have been shown to be pathogenic in a growing number of viral diseases since the COVID-19 pandemic. They were first shown in 2020–2021 to cause ∼15% of cases of life-threatening COVID-19 pneumonia (32, 34, 35, 36, 37, 38, 39, 40, 41, 42, 43, 44, 45, 46, 47, 48, 49, 50, 51, 52, 53, 54, 55, 56, 57, 58, 59, 60, 61, 62, 63, 64, 65, 66, 67, 68), then ∼5% of cases of life-threatening seasonal influenza pneumonia (73) and ∼25% of cases of life-threatening Middle East respiratory syndrome pneumonia (74). It soon became apparent that these auto-Abs can also underlie life-threatening arboviral infections (Fig. 2). They are strong determinants of severe adverse reactions to the YFV live-attenuated vaccine, WNV encephalitis, TBE, POWV encephalitis, severe USUV disease, and RRV disease (87, 88, 99, 103, 108). With the exception of severe adverse reactions to the YFV-17D vaccine, the other arboviral diseases follow the transmission of an orthoflavivirus (WNV, TBEV, POWV, and USUV) or an alphavirus (RRV) from animals to humans by mosquitoes (WNV, USUV, and RRV) or ticks (TBEV and POWV). All these viruses cause neurotropic disease, except for RRV, which is arthritogenic. The cellular and molecular mechanisms by which AAN-I-IFN underlie these severe arboviral infections remain to be clarified. Do type I IFNs normally block viral infection in the skin, blood, or brain? Which type I IFN–induced genes encode proteins that normally restrict viral infection in vivo? A forward genetic approach might help tackle these important questions (131). The AAN-I-IFN precede infection. The proportion of severe cases explained by these auto-Abs varies for each arboviral disease, but it is always high and causality is, therefore, almost certain, particularly given the very high ORs reported for infections in studies with a sufficiently large sample size. These auto-Abs have been shown to underlie severe cases for all arboviral infections studied to date. Future studies should focus on other arboviral infections, including those due to ZIKV, DENV, and CHIKV, to determine whether AAN-I-IFN are also determinants of severe infection with these viruses. Genetic defects of type I IFN immunity may be the cause of severe arboviral infections in people who do not carry AAN-I-IFN, warranting future investigations as well (131).

These discoveries have major clinical and public health implications. First, these auto-Abs have been found worldwide, with a prevalence of about 0.5% in individuals under the age of 65 years and 5% in those over the age of 70 years due to germline (mostly in the young) or somatic (mostly in the elderly) genetic defects likely impairing thymic tolerance to self (I-IFN) (132, 133, 134, 135, 136) (Fig. 2). We can therefore estimate that they are carried by over 100 million people globally. They may explain other severe viral illnesses beyond respiratory viral diseases and arboviral diseases. It has become important to determine the range of severe viral diseases potentially due to circulating AAN-I-IFN and the proportion of cases explained by AAN-I-IFN. Meanwhile, these auto-Abs should be systematically sought in any patient with unexplained severe arboviral disease. We recently developed a cheap, rapid test for use on whole blood to detect inherited or acquired deficits of type I IFN in a clinical laboratory (137). Benjamin G. Hale in Zurich has developed a rapid sensitive test to screen for auto-Abs against type I IFNs neutralizing concentrations as low as 10 pg/ml (133, 138, *Preprint*). The identification of a deficiency of type I IFN immunity might improve the management of some patients who could benefit from the administration of type I IFNs not neutralized by their auto-Abs. High doses of type I IFNs might even compensate for a lack of the same specific type I IFN subtypes neutralized by the patient’s auto-Abs, as shown in patients with auto-Abs against granulocyte-macrophage colony-stimulating factor (139, 140, 141, 142). Importantly, people with these auto-Abs should not be vaccinated with live-attenuated viral vaccines. It is tempting to speculate that the recent adverse reactions reported in elderly individuals vaccinated with a live-attenuated CHIKV vaccine were also due to auto-Abs against type I IFNs (press release, https://sante.gouv.fr/actualites/presse/communiques-de-presse/article/les-autorites-sanitaires-retirent-les-personnes-de-65-ans-et-plus-des-cibles-de). Finally, people living in regions in which such arboviruses are endemic, or people planning to travel to such regions, should potentially be tested for these auto-Abs. They could then adapt their behavior and use specific measures to avoid being bitten by arthropods. This is important, given the absence of human vaccines or specific antiviral treatments for most arboviruses.
